# Air-conditioner cooling towers as complex reservoirs and continuous source of *Legionella pneumophila* infection evidenced by a genomic analysis study in 2017, Switzerland

**DOI:** 10.2807/1560-7917.ES.2019.24.4.1800192

**Published:** 2019-01-24

**Authors:** Daniel Wüthrich, Sylvia Gautsch, Ruth Spieler-Denz, Olivier Dubuis, Valeria Gaia, Jacob Moran-Gilad, Vladimira Hinic, Helena MB Seth-Smith, Christian H. Nickel, Sarah Tschudin-Sutter, Stefano Bassetti, Monika Haenggi, Peter Brodmann, Simon Fuchs, Adrian Egli

**Affiliations:** 1 Division of Clinical Microbiology, University Hospital Basel, Basel, Switzerland; 2 Applied Microbiology Research, Department of Biomedicine, University of Basel, Basel, Switzerland; 3 Swiss Institute of Bioinformatics, Basel, Switzerland; 4 State Laboratory Basel-City, Basel, Switzerland; 5 Department of Health, Medical Services, Canton of Basel-Stadt, Basel, Switzerland; 6 Viollier, Allschwil, Switzerland; 7 National Reference Center for Legionella, Department of Laboratory medicine, Ente Ospedaliero Cantonale, Bellinzona, Switzerland; 8 Department of Health Systems Management, School of Public Health, Faculty of Health Sciences, Ben-Gurion University of the Negev, Beer-Sheva, Israel; 9 Public Health Services, Ministry of Health, Jerusalem, Israel; 10 Division of Emergency Medicine, University Hospital Basel, Basel, Switzerland; 11 Division of Infectious Diseases and Hospital Epidemiology, University Hospital Basel, Basel, Switzerland; 12 Division of Internal Medicine, University Hospital Basel, Basel, Switzerland; 13 Department of Health, Medical Services, Canton of Basel-Country, Liestal, Switzerland

**Keywords:** whole genome sequencing, WGS, cooling tower, Legionella pneumophila, L. pneumophila, Legionnaires’ disease, outbreak, Switzerland

## Abstract

**Introduction:**

Water supply and air-conditioner cooling towers (ACCT) are potential sources of *Legionella pneumophila* infection in people. During outbreaks, traditional typing methods cannot sufficiently segregate *L. pneumophila* strains to reliably trace back transmissions to these artificial water systems. Moreover, because multiple *L. pneumophila* strains may be present within these systems, methods to adequately distinguish strains are needed. Whole genome sequencing (WGS) and core genome multilocus sequence typing (cgMLST), with their higher resolution are helpful in this respect. In summer 2017, the health administration of the city of Basel detected an increase of *L. pneumophila* infections compared with previous months, signalling an outbreak.

**Aim:**

We aimed to identify *L. pneumophila *strains populating suspected environmental sources of the outbreak, and to assess the relations between these strains and clinical outbreak strains.

**Methods:**

An epidemiological and WGS-based microbiological investigation was performed, involving isolates from the local water supply and two ACCTs (n = 60), clinical outbreak and non-outbreak related isolates from 2017 (n = 8) and historic isolates from 2003–2016 (n = 26).

**Results:**

In both ACCTs, multiple strains were found. Phylogenetic analysis of the ACCT isolates showed a diversity of a few hundred allelic differences in cgMLST. Furthermore, two isolates from one ACCT showed no allelic differences to three clinical isolates from 2017. Five clinical isolates collected in the Basel area in the last decade were also identical in cgMLST to recent isolates from the two ACCTs.

**Conclusion:**

Current outbreak-related and historic isolates were linked to ACCTs, which form a complex environmental habitat where strains are conserved over years.

## Introduction

*Legionella pneumophila* (Lp) causes Legionnaires’ disease (LD), a severe infection of the respiratory tract. LD was first described in 1976 after an outbreak at an American legion convention due to a contaminated air-conditioning system [1]. In that outbreak, 182 persons were infected and 29 (16%) died [1]. Since then, Lp has been considered an important threat to public health. The European Legionnaires’ disease Surveillance network (ELDSNet) reported, that between 2011 and 2015 across 29 European countries a total of 30,532 LD cases were documented, whereas the incidence rose from 0.97 (2011) to 1.30 (2015) per 100,000 inhabitants. Most LD cases are community-acquired and affect people aged 50 years or older, with mortality rates around 10% [2]. In Switzerland during 2017, 492 cases of LD were reported [3] with anincidence of 5.81 per 100,000 inhabitants.

Infections with Lp are acquired via inhalation of contaminated aerosolised water [4]. Various environmental sources are known, such as showers [5,6], hot tubs, fountains, dishwashers [7], hot water tanks, larger plumbing systems [8] and air-conditioner cooling towers (ACCT) [9-12]. 

A given environmental source can host several types of Lp strains, which, in some cases, can enter amoeba biofilms [4] leading to low mutation rates and a high conservation of genomic diversity. As a consequence, traditional typing methods such as serotyping, pulsed-field electrophoresis (PFGE), and sequence-based typing (SBT) do not provide sufficient resolution to trace outbreaks to individual sources. In addition, certain Lp clonal complexes of clinical relevance (e.g. ST1) are spread worldwide, and respective isolates are so similar, that SBT cannot distinguish them [13]. This renders SBT insufficient for typing Lp for public health purposes. On the other hand, different isolates of a specific clonal complex may have a limited number of single nt polymorphisms (SNPs) (e.g. ST1: 121 SNPs), which are detectable by whole genome sequencing (WGS) to allow their discrimination.

The ability of WGS to deliver complete genomic information [14], thereby conferring higher-resolution, has made it the gold standard for typing Lp isolates. Moreover, investigations of LD incidents in a fast and automatic manner have recently been facilitated by a core genome multilocus sequence typing (cgMLST) scheme based on WGS data [15]. Beside single outbreak investigations, WGS-based typing data also support comparison across studies [16]. Nevertheless, many recent reports on Lp using WGS have mainly focused on single outbreaks [5,8,12,17,18]. These studies also did not assess the complexity of environmental sources in great detail, whereby the sampling strategy of the environmental isolates and the diversity of strains in the sources remain unclear.

Based on epidemiological evidence, ACCTs are suspected to be a considerable source of outbreaks [17,19-21], yet the *Legionella* populations within have not been thoroughly described. To clarify the transmission mechanism of Lp, which in turn guides appropriate control measures, it is important to understand the environmental complexity of Lp populations (e.g. genomic diversity, exchange between populations) and relate this to data from outbreak-related clinical isolates. The goal of this study was to extend our knowledge of the role of environmental Lp sources, such as ACCTs and water supply, during an outbreak, or over a prolonged time period. Therefore, we studied clinical isolates from the city of Basel and surrounding areas during an outbreak in 2017 and compared these to isolates originating from water pipes and ACCTs by applying WGS. We also sequenced clinical isolates that were collected since 2003. With these data we attempt to identify links between Lp populations within ACCTs, and outbreak-related and historical clinical isolates.

## Methods

### Setting

In Switzerland, all positive Lp cases have to be reported to the federal office of health by law [3], which is followed by an environmental risk assessment. Briefly, cases clinically suspected of respiratory tract infection get screened using a urinary *Legionella* antigen testing according to the manufacturer (BinaxNOW from Alere, which detects serotypes 1–14 or Sofia Legionella FIA from Quidel, which detects serotype 1; San Diego, United States). In the case of a positive Lp result, the treating physician is contacted to report the result and send respiratory material for culture-based detection and subsequent typing of the Lp isolate.

We cultured 34 strains from humans. Four cultured isolates (isolate ID: NMB001740, NMB001739, NMB001863, NMB001758) of Lp serotype 1 obtained in the time period of the outbreak and the specific city district associated with the outbreak were available for WGS analysis. As non-outbreak controls, we included four serotype 1 isolates from the same time period, but different geographical areas including the neighbouring cantons (n = 3) and another city district of Basel (n = 1). Furthermore, we included 26 historic isolates collected between 2003 and 2016 in the canton of Basel-city and the neighbouring cantons. Additionally, we used 60 Lp isolates from the local water supply chain and ACCTs within the area of the outbreak (up to 29 isolates per location). The details of the samples are listed in Supplementary Table S1.

### Origins, culture and serogroup identification of human isolates

Respiratory materials such as sputum, tracheal secretion and bronchioalveolar lavages, were cultured for a maximum of 10 days at 36 °C under 5% CO_2_ using buffered media with polymyxin B, anisomycin and alpha-ketoglutarate (BMPA from Thermoscientific, Reinach, Switzerland) and standard 5% sheep blood agar (bioMérieux, Lyon, France). Culture plates were daily checked for growth and suspected colonies were identified using matrix assisted laser desorption ionization-time of flight (MALDI-TOF) mass spectrometry (Microflex system, Bruker, Bremen, Germany). Lp isolates were further separated into serogroup 1 or 2–14 (Legionella latex test, Oxoid (Pratteln, Switzerland)). The historic isolates were obtained from the strain collection of the University Hospital Basel and respective serogroups were determined in the same way.

### Origins, culture and serogroup identification of environmental isolates

Water samples (1,000 mL) from suspected environmental sources (tap water sources/plumbing systems, ACCTs) were filtered and cultured directly and after filtration without treatment, after acid treatment and after heat treatment following the International Standard ISO 11731:2017 ‘Water quality – Enumeration of *Legionella*’. The isolates were cultured aerobically for a maximum of 10 days at 37 °C using the selective media buffered charcoal yeast extract agar with polymyxin B, anisomycin and cephamandol (BMPA from Oxoid, Pratteln, Switzerland), MWY (buffered charcoal yeast extract agar with glycin, polymyxin B, anisomycin, vancomycin, bromothymol blue and bromocresol purple; Oxoid, Pratteln, Switzerland) and GVPC (buffered charcoal yeast extract agar with glycin, vancomycin, polymyxin B and cycloheximide; Oxoid, Pratteln, Switzerland). Culture plates were checked every 2–3 days for growth and suspected colonies were identified by subculture on buffered charcoal yeast extract agar (BCYE-agar; Oxoid, Pratteln, Switzerland) with L-cysteine and on standard 5% sheep blood agar (bioMérieux, Lyon, France). Isolates showing no growth on cysteine-free blood agar were considered as *Legionella* and further identified by agglutination and separated into serogroup 1 or 2–14 (Legionella latex test, Oxoid (Pratteln, Switzerland)). Finally, colony forming units of Lp per mL and per 1,000mL of water sample were determined.

### Whole genome sequencing of bacterial isolates and bioinformatic analysis

From both clinical and environmental isolates, we included each morphotype to WGS analysis. DNA from cultured isolates was extracted using a robotic system (EZ1 Advanced XL, Qiagen (Venlo, Netherlands)). WGS sequencing was performed using a MiSeq Illumina platform (accredited with ISO 17025 norm) with 2x 300nt paired-end sequencing as previously described [22]. The resulting reads were de novo assembled using Unicycler [23] (version 0.4.4) and the assemblies (assembly statistics are listed in Supplementary Table S2) used for cgMLST analysis performed with Ridom SeqSphere Software (version 4.1.9) using the recently published cgMLST scheme [15]. All isolates had at least a mean coverage of 90-fold. All genomes sequenced for this study were submitted to GenBank (see accession numbers Supplementary Table S2).

All available Lp genome assemblies were downloaded from the National Center for Biotechnology Information (NCBI, December 2017, 539 genomes). The assemblies were re-annotated using Prokka (version 1.12) [24] for consistency, and phylogenetic analysis that was based on the core genome alignment was performed using Roary (version 3.11.2) [25] and FastTree (version 2.1) [26]. The phylogenetic tree was visualised using iTOL [27]. Whole genome comparison (SNP-calling) was performed using BWA (version 0.7.17) [28] and Pilon (version 1.22) [29].

## Results

### Description of *Legionella pneumophila* outbreak in Basel 2017

In 2017, the weekly number of LD cases in the city of Basel appeared to increase from May to August (Supplementary Figure S1). In this city, the overall incidence per 100,000 inhabitants increased from 4.66 to 15.02 between 2016 and 2017 [3] (Supplementary Figure S2). Although, no active case finding strategy was developed, the health administration of the city of Basel performed a detailed epidemiological investigation using a standardised questionnaire to assess potential risk factors for *Legionella* exposure for all infected patients (Supplement S1, Supplement S2). Based on the investigation results, including the place of residence of the patients, a spatial and temporal cluster of Lp serotype 1 infected patients in a particular city district was found. A secondary investigation with more specific questions about epidemiological risk factors and places visited was performed. Thereby, the area and particular exposures could be even further specified. Interestingly, ACCTs were found in the vicinity of some of the patients’ homes.

### Whole genome sequencing typing of human isolates

The WGS-based cgMLST comparison showed that three of the four putative outbreak isolates had the same cgMLST type (cluster type 228), with no allelic differences (0/1521). Therefore, these patients were infected with the same strain. The other five clinical (including the one outbreak isolate, and the four non-outbreak isolates) isolates from 2017 (cluster types are listed in Supplementary Table S1) showed more than 90 allelic differences to the cluster of three samples, indicating that these patients were infected with other strains.

### Investigation of environmental sources

In order to identify a possible source of infection for the three patient isolates sharing the same cgMLST type, we sampled water sources from plumbing systems in close proximity to their respective place of residence. We identified four different locations contaminated with Lp (Supplementary Table S1). Colonies with various morphotypes were selected. The investigated locations contained Lp serotypes 1 and 2–14. Because patients had been tested with a serotype 1 specific urinary antibody test in 2017 only serotype 1 clinical isolates were available for comparison.

Based on the epidemiological assessment of the outbreak cluster patients, we suspected eight ACCTs as possible sources of *Legionella*. As there is no cooling tower registry for the city of Basel, we used the epidemiological risk assessment to identify the most likely towers in close distance for the sampling. Material from two of these eight sampled ACCTs yielded growth of Lp. In the two ACCTs various morphotypes could be detected on the culture plates, including serotypes 1 and 2–14, all of which were included into the WGS-analysis. Quantitative analysis of *Legionella* in the water samples from these contaminated ACCTs reached up to 5.8 million colony forming units per litre (Supplementary Table S1), which reflects a high pathogen density. The water flow and aerosolisation associated with functions of an ACCT are shown in Supplementary Figure S3.

### Whole genome sequencing analysis of clinical and environmental isolates

WGS was performed on 37 isolates from the two contaminated ACCTs and 23 isolates from the four plumbing systems contaminated with Lp (Supplementary Table S1). Isolates were selected based on differing morphotypes from the different isolation sites. In addition, we included 26 historic clinical isolates from the strain collection of the University Hospital Basel, collected since 2003 from the city of Basel and surrounding area.

A total of 94 clinical and environmental isolates were analysed using cgMLST. The overall diversity throughout all isolates was very high, covering more than one thousand allelic differences (Figure), producing 13 closely related complexes (≤ 10 allelic differences) and also 15 strains without close relation to other isolates (Figure).

**Figure:**
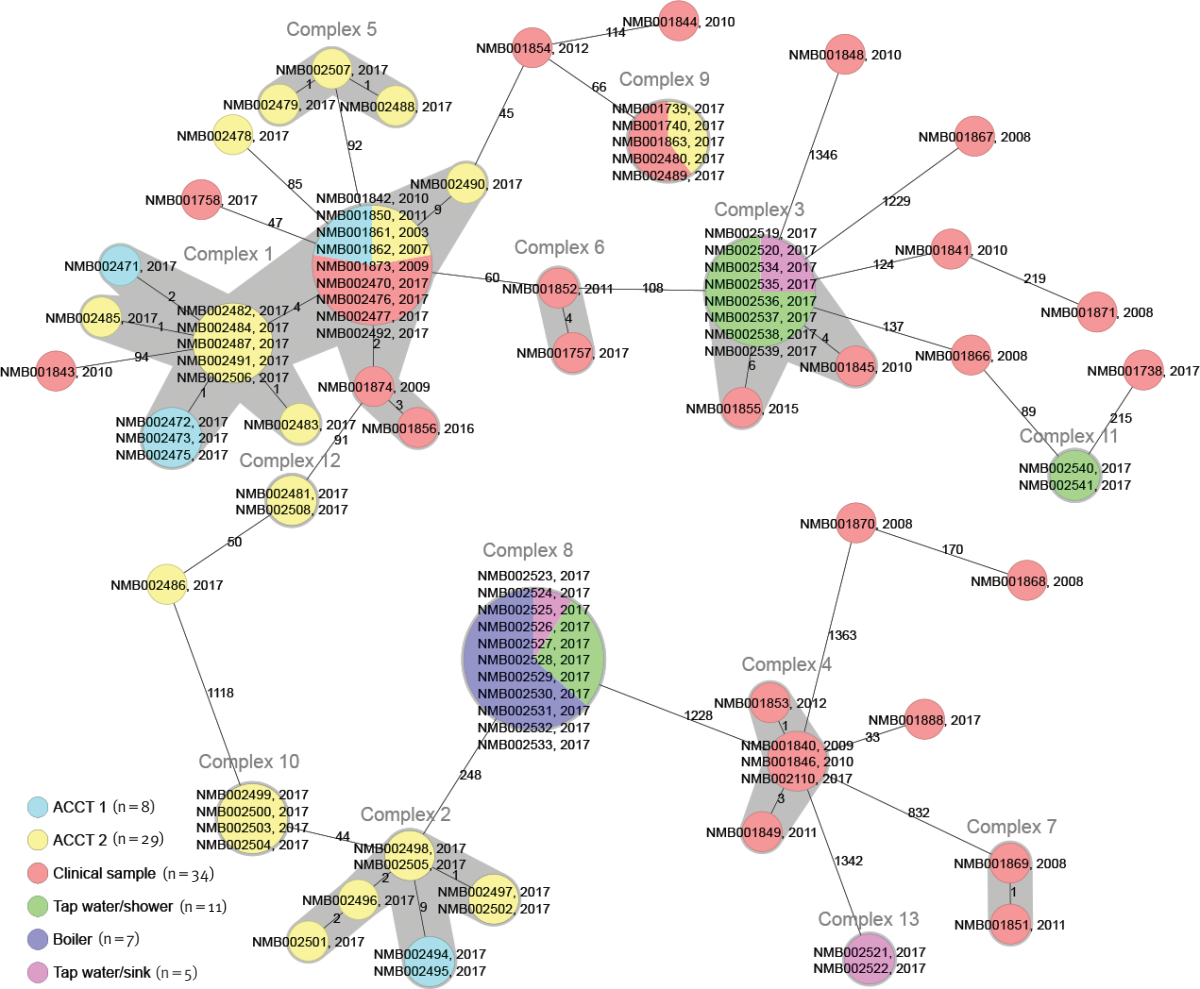
Allelic differences between the *Legionella pneumophila* strains recovered in clinical and environmental isolates, Switzerland, 2003–2017 (n = 94 isolates)

ACCT: air-conditioner cooling towers; cgMLST: core genome multilocus sequence typing.

The circles represent sequenced strains. The circles are coloured according the isolation source. The numbers next to the lines connecting two circles indicate the number of allelic differences. Strains that have no allelic differences are listed in the same circle. Strains with 10 or less allelic differences are clustered into complexes and connected with a grey background. The analysis is based on the published cgMLST scheme [15] for *Legionella pneumophila* using 1,521 allelic loci. The last four numbers next to the isolate ID indicate the isolation year.

Our first analysis focused on the environmental samples, which were found within ten complexes (Figure). ACCT-derived isolates can be found within six different complexes, while all environmental isolates recovered from tap water and plumbing sources (Figure) were found in four different clusters. Complexes 1 and 2 contain isolates from the two ACCTs sites. Most interestingly, isolates within complex 1 originated from both ACCTs, including some isolates from both ACCTs with no allelic differences.

The comparison of the environmental and clinical samples showed that the three identical clinical outbreak isolates are closely associated with two isolates from a single ACCT (Figure, ‘complex 9’), showing no allelic differences in the cgMLST analysis. This analysis was complemented by a whole genome based variant calling approach for increased typing resolution. This approach revealed a variability of only 5 SNPs within that cluster, further highlighting the close relatedness (data not shown). We also analysed all serogroup 1 strains of the ACCT using SBT that showed that all strains were ST36 (Philadelphia). Furthermore, we also found that five historic isolates, sampled between 2003 and 2011, were within complex 1, and showed no allelic differences to four environmental isolates from the ACCTs (Figure, ‘complex 1’). Therefore, we concluded that this environmental strain, recovered during the current investigation, has been causing infections over the past decades. In total, we observed that 12 clinical isolates (historic and 2017) had 10 or fewer allelic differences compared with the closest related environmental isolate.

Nevertheless, not all historic or current clinical isolates could be linked to the sampled environmental isolates. Interestingly, we found that of these 22 clinical isolates (17 historic and five from 2017) nine are found in three complexes (Figure, complexes 4,6,7). Especially interesting is complex 4, as it contains five clinical isolates from 2009 to 2017. The remaining 13 clinical isolates are not closely related to any other isolate. To investigate potential origins of these 22 patient strains without connection to environmental isolates, we accessed 539 Lp genomes from the public NCBI database, reflecting a global strain collection. We performed phylogenetic analysis of all sequenced strains from Basel and the genomes from NCBI that were isolated in 17 different countries (clinical and environmental samples). In order to handle the high number of genomes (n = 633), we compared the strains using a core genome alignment-based phylogeny. The analysis showed that all 22 strains without links to environmental isolates are closely related to isolates from other European countries (Supplementary Figure S4).

## Discussion

In this study, we have shown, based on WGS and cgMLST analysis, that clinical isolates associated with the outbreak of 2017 in the city of Basel are genetically related to ACCT-derived isolates. This finding supports that ACCTs can act as a source of *Legionella* infection, as suspected in previous studies [19,20]. An important finding of our study is the broad genetic diversity of environmental isolates across the city. Although the isolates sampled from two ACCTs were found to be very closely related (complex 1 and 2), the findings clearly highlight the need to sample a broad range of environmental reservoirs in an outbreak setting in order to identify the causal source. Due to the diversity within these environmental reservoirs, we believe that shotgun metagenomics [30] could provide more information than WGS on selected isolates, as the latter might overlook important strains. However, this approach would necessitate the use of appropriate and maybe newly developed bioinformatics tools that allow the differentiation of strains in metagenomics samples [31,32].

We have demonstrated that identical environmental isolates can be found in different sampling locations, potentially indicating a complex environmental network. As there was no direct water pipe connection between the two contaminated ACCTs in this study that are ca 500 m apart, our current assumption is that the release of contaminated aerosols not only leads to human exposure, but also facilitates the exchange of *Legionella* populations between ACCTs. Some previous studies have attempted to characterise Lp populations in ACCTs. In 103 water samples from 50 ACCTs collected over five years in Turkey (1996–2000), relatively stable serotype distributions with 44% serotype 1 have been described [33]. Another study used 16S sequencing to study the *Legionella* species dynamics within cooling towers and found that Lp can outcompete other *Legionella* species [30]. However, to date no high-resolution analysis of Lp within ACCTs has been conducted. Our findings highlight the potential of (i) a complex environmental network and (ii) suggest that decontaminated ACCTs (the decontamination automatics were defect in the observed ACCTs) can be potentially recolonised by contaminated aerosols from other ACCTs. This information could be used to influence the design of ACCTs (Supplementary Figure S3) and strategies in the control of potential outbreak sources [34,35]. Our study already had a real-life effect, as for the two contaminated ACCTs, the maintenance procedure for decontamination was corrected after our findings.

The diversity of isolates within one environmental *Legionella* population, as shown by the WGS data, is also remarkable. We found isolates from the same populations that are separated by more than one thousand allelic differences. Interestingly, only environmental isolates from complex 1, 3 and 9 were connected to clinical samples (Figure). The cgMLST results indicates that subclones of the same ST (e.g. ST36) seem to have enhanced potential for causing infection, as out of the 15 cluster types that we found in the environmental samples, only cluster type 177 and 228 also comprised the clinical samples.

The inclusion of previously collected isolates from the strain collection of the University Hospital Basel allowed us to increase the sample size, and also to link historic cases to environmental contamination. The transmission from ACCTs appears not to be a rare event that is limited to the outbreak from 2017. We were able to connect some clinical isolates found in ACCTs to clinical infections that occurred almost a decade apart and the strains can still be found in the ACCT (Figure, ‘complex 1’). However, this is not limited to only one event, we found several cases of closely related clinical isolates that were isolated in different years (Figure). We concluded that these are conserved *Legionella* strains in environmental sources, that lead to infections over several years and that these environmental sources form a complex network. This is in agreement with another study, where the same strains were found over several years [8]. We assume strains are conserved over the years in biofilms [4]. Unfortunately, no historical environmental samples were available to test our hypothesis.

While our findings provide more insight into potential links between complex environmental Lp reservoirs and LD, this study has several limitations. First, we only had a limited number of isolates available, in particular isolates were not obtained from all outbreak-related patients. Although a total of 94 isolates were included, the study would certainly have further benefitted from a higher sampling density of environmental and clinical isolates. Often clinical isolates cannot be collected, as patients with a positive antigen test in urine samples will receive treatment and no culture isolation from respiratory material is performed. Clearly, physicians should be aware of the importance of *Legionella* culture and WGS-based typing for public health reasons. In addition, the sensitivity of culture-based methods for *Legionella* detection is somewhat limited [36]. Another limitation was that unfortunately, we did not have historical samples from the environment that could match historical clinical samples. Finally, we were only able to sample two ACCT sites, although the exchange of strains between both systems could be documented, more systems should be sampled and analysed in the future.

In conclusion, we showed that contaminated ACCTs are an important threat to public health. WGS played a crucial role in this study, as it allowed the high-resolution typing and therefore demonstrated the value of this technique in clinical microbiology. In particular, the potential that environmental systems can form a complex network without having a direct water supply connection is an important finding. Finally, we have shown that strains are conserved and cause infections over decades.

## Supplementary Data

46000 Supplement S1

## Supplementary Data

156000 Supplement S2

## Supplementary Data

15000 Supplementary Figure S1

## Supplementary Data

15000 Supplementary Figure S2

## Supplementary Data

55000 Supplementary Figure S3

## Supplementary Data

251000 Supplementary Figure S4

## Supplementary Data

15000 Supplementary Table S1

## Supplementary Data

17000 Supplementary Table S2
