# Supplementary material for: Air-conditioner cooling towers as complex reservoirs and continuous source of Legionella pneumophila infection evidenced by a genomic analysis study in 2017, Switzerland
Source: Euro Surveill. 2019 Jan 24;24(4):1800192. doi: 10.2807/1560-7917.ES.2019.24.4.1800192 (PMC6351994; doi:10.2807/1560-7917.ES.2019.24.4.1800192)
Supplement: Supplement S1 [file 1800192_SupplementS1.pdf]

This supplementary material is hosted by Eurosurveillance as supporting information alongside the article “Air-conditioner cooling towers as complex reservoirs and continuous source of Legionella pneumophila infection evidenced by a genomic analysis study in 2017, Switzerland” on behalf of the authors who remain responsible for the accuracy and appropriateness of the content. The same standards for ethics, copyright, attributions and permissions as for the article apply. Eurosurveillance is not responsible for the maintenance of any links or email addresses provided therein.

Basic data collected by the medical services of the City of Basel

Personal data:
- Name
- Date of Birth
- Gender
- Place of living: address
- Contact information: phone
- Nationality
- Current profession: what and where
- Past profession: what, where, and until when
- Date of getting disease
- Hospitalisation: yes/no, if yes exact dates

Known baseline diseases and risk factors
- Respiratory tract e.g. COPD
- Cancer
- Diabetes mellitus
- Immunosuppression: acquired and medication
- Transplantation
- Cardiac disease
- Others diseases

- Drinking: yes/no, if yes: amount per day
- Smoking: yes/no, if yes: amount per day

Travel related questions
Did you travel within the last 2 weeks?
- overnight stay: yes/no, if yes: duration
- own apartment: yes/no, if yes: where
- hotel: yes/no, if yes: where and room number
- camping: yes/no, if yes: where

Living related questions
- single house: yes/no
- apartment house: yes/no, if yes: size of the building, amount of apartments, amount of floors, empty apartments
- Contact for apartment houses
- construction year of the building
- plumbing system: construction year, type of heating, water temperature, boiler system

Potential sources of infections at place of living
- Water probing sites: amounts and locations
- Showers: used and unused
- Inhalation equipment: yes/no
- Air conditioner: yes/no
- Humidifier: yes/no
- Fountains: yes/no

- Garden: yes/no, if yes: rain collection, sprinkler, garden hose

Potential sources of infections close to place of living
- Water spray: yes/no
- Air conditioning cooling towers: yes/no
- Fountains: yes/no

Work related questions
- Name of company
- Location
- Do you work with water? Contact with water?

Potential sources of infection at the working place
- Water-tabs: amount, used/unused
- Showers: amount, used/unused
- Inhalation equipment: yes/no
- Air conditioner: yes/no
- Humidifier: yes/no
- Aerosol equipment: yes/no
- Room fountain: yes/no
- Garden: yes/no, if yes: rain collection, sprinkler, garden hose

Potential infections sources close to the place of working
- Aerosol equipment: yes/no
- Air conditioning cooling towers: yes/no
- Fountains: yes/no

Potential other sources
Did you visit any of the places within the last 2 weeks? If yes: where?
- Nursing home: yes/no
- Aquarium: yes/no
- Car washing facility: yes/no
- Shopping center: yes/no
- Gym/Spa: yes/no
- Hair stylist: yes/no
- Market with aerosol equipment: yes/no
- Sauna/Wellness facility: yes/no
- Hospital: yes/no
- Sport facility: yes/no
- Swimming or whirlpool: yes/no
- Dentist: yes/no

Do you go walking on a regular basis? If yes: Where?
