# Supplementary material for: Air-conditioner cooling towers as complex reservoirs and continuous source of Legionella pneumophila infection evidenced by a genomic analysis study in 2017, Switzerland
Source: Euro Surveill. 2019 Jan 24;24(4):1800192. doi: 10.2807/1560-7917.ES.2019.24.4.1800192 (PMC6351994; doi:10.2807/1560-7917.ES.2019.24.4.1800192)
Supplement: Supplement S2 [file 1800192_SupplementS2.pdf]

This supplementary material is hosted by Eurosurveillance as supporting information alongside the article "Air-conditioner cooling towers as complex reservoirs and continuous source of Legionella pneumophila infection evidenced by a genomic analysis study in 2017, Switzerland" on behalf of the authors who remain responsible for the accuracy and appropriateness of the content. The same standards for ethics, copyright, attributions and permissions as for the article apply. Eurosurveillance is not responsible for the maintenance of any links or email addresses provided therein.

[redacted]

Persönliche Daten

Name	Nachname	Vorname	Vorname
Geburtsdatum	Geburtsdatum	Geschlecht	Geschlecht
Strasse	Adresse	Ort	Ort
Kanton	Kanton	PLZ	PLZ
Telefonnummer:	Telefonnummer		
Nationalität	CH	Andere Nationalität? Welche?	______
Beruf (derzeit)	______	Rentner / Pensionär	ja
Beruf (früher)	______	Bis wann?	______
Datum der Erkrankung	_____ / _____ / _______		
Spitalaufenthalt von	_____ / _____ / _______	bis	_____ / _____ / _______

**Vorbestehende Erkrankungen / Risikofaktoren**	**ja**	**nein**		
Atemwegserkrankung				
Krebs				
Diabetes mellitus				
Eingeschränktes Immunsystem				
Immunsuppressive Medikamente				
Transplantation				______
Herzkrankheit				______
Andere			welche?	______
Alkoholkonsum			Gläser pro Tag - Anzahl	______
Rauchen			Zigaretten pro Tag - Anzahl	______

Reisen:

Hatten Sie in den 2 Wochen vor und bis zu Ihrer Erkrankung eine Reise unternommen?

	**ja**	**nein**	Wenn ja, wohin? Welche Adresse?
Mit Übernachtung?			
Dauer?			
Ferienhaus			______
			______

[redacted]

Hotel und ZimmerNr.			1
			2
			3
Zeltplatz/Camping			

Wohnort

	ja	nein
**Einfamilienhaus**		
**Mehrfamilienhaus**		

Anzahl Wohnungen ________ Anzahl Stockwerke ________

Anzahl der bewohnten Wohnungen ________

Unbewohnte Wohnungen; seit wann? ________

Baujahr des Hauses ________

Wasserleitungssystem

Baujahr Heizung ________ Renovationsarbeiten im Jahr: ________

Art des Heizungssystems ________

Baujahr Wasserleitungssystem ________ Renovationsarbeiten im Jahr: ________

Wasser-Temperatur Vorlauf ________ am Hahnen ________

Boiler ________ Letzte Entkalkung/Reinigung ________

Mögliche Infektionsquellen am Wohnort

Wasserentnahmestellen Anzahl ________

Davon in Gebrauch - Anzahl ________ nicht in Gebrauch - Anzahl ________

Duschen Anzahl ________

Davon in Gebrauch ________ nicht in Gebrauch - Anzahl ________

**Befindet sich am Wohnort oder benutzen Sie**	**ja**	**nein**	**?**	Wenn ja, wo?
Inhalationsgerät				
Klimaanlage				
Luftbefeuchter				
Sprühflaschen				
Zimmer-, Zier-, Springbrunnen				
**Garten / Aussenanlagen**	**ja**	**nein**	**?**	Wenn ja, wo?

[redacted]

Regenauffangbecken				
Sprühanlage				
Wasserschlauch				

Mögliche Infektionsquellen in der Nähe des Wohnorts

**Gibt es in der Nähe:**	**ja**	**nein**	**?**	Wenn ja, wo?
Anlagen mit Sprühwasser				
Kühltürme				
Springbrunnen				

Mögliche Infektionsquellen am Arbeitsplatz (nur bei Berufstätigkeit ausfüllen)

Name des Betriebes ______________________

Strasse ______________________ Ort ______________________

Kanton ______________________

Tätigkeit, bei der Sie am Arbeitsplatz mit Wasser in Kontakt kommen ______________________

Wasserspender / Hahnen Anzahl ________

Davon in Gebrauch - Anzahl ________ nicht in Gebrauch - Anzahl ________

Duschen Anzahl ________

Davon in Gebrauch ________ nicht in Gebrauch - Anzahl ________

**Befindet sich am Arbeitsplatz oder wird da benutzt**	**ja**	**nein**	**?**	Wenn ja, wo?
Inhalationsgerät				
Klimaanlage				
Luftbefeuchter				
Sprühflaschen				
Zimmer-Springbrunnen				
**Garten / Aussenanlagen**	**ja**	**nein**	**?**	Wenn ja, wo?
Regenauffangbecken				
Sprühanlage				
Wasserschlauch				

Mögliche Infektionsquellen in der Nähe des Arbeitsplatzes

**Gibt es in der Nähe:**	**ja**	**nein**	**?**	Wenn ja, wo?

[redacted]

Anlagen mit Sprühwasser				
Kühltürme				
Springbrunnen				

Weitere mögliche Infektionsquellen

Haben Sie in den 2 Wochen vor Ihrer Erkrankung einen der folgenden Orte besucht oder haben Sie sich dort aufgehalten?

	ja	nein	?	Wenn ja, wo? Welche Adresse?
Alters- und Pflegeheim				
Aquarium				
Autowaschanlage				
Einkaufszentrum/ -laden				
Fitnesscenter				
Friseur				
Krankengymnastische Praxis				
Markt mit Sprühanlagen				
Sauna				
Spital				
Sportanlage oder Sportverein				
Thermalbad, Schwimmbad, Whirlpool				
Zahnarzt				

[redacted]

Gehen Sie regelmässig spazieren? Wo waren Sie in den 2 Wochen vor der Legionellose spazieren?

1 ______

2 ______

3 ______

4 ______

Hausverwaltung

Zuständiger Verwalter

Adresse
