# Supplementary material for: Air-conditioner cooling towers as complex reservoirs and continuous source of Legionella pneumophila infection evidenced by a genomic analysis study in 2017, Switzerland
Source: Euro Surveill. 2019 Jan 24;24(4):1800192. doi: 10.2807/1560-7917.ES.2019.24.4.1800192 (PMC6351994; doi:10.2807/1560-7917.ES.2019.24.4.1800192)
Supplement: Supplementary Figure S3 [file 1800192_SupplementaryFigureS3.pdf]

This supplementary material is hosted by Eurosurveillance as supporting information alongside the article “Air-conditioner cooling towers as complex reservoirs and continuous source of Legionella pneumophila infection evidenced by a genomic analysis study in 2017, Switzerland” on behalf of the authors who remain responsible for the accuracy and appropriateness of the content. The same standards for ethics, copyright, attributions and permissions as for the article apply. Eurosurveillance is not responsible for the maintenance of any links or email addresses provided therein.

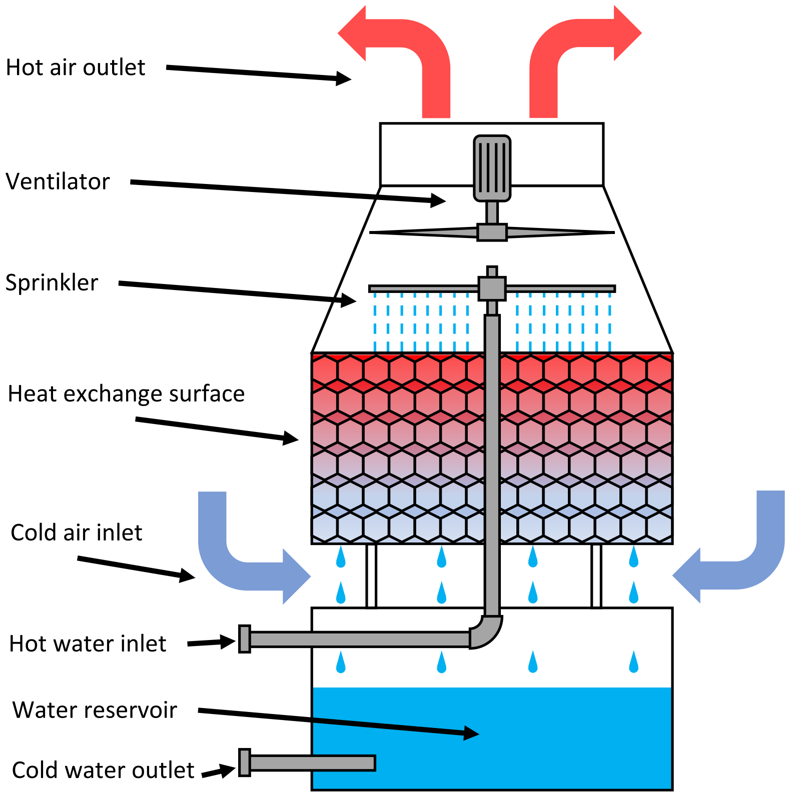

Figure S3: Schematic representation of an air-conditioners cooling tower. The hot water from with the air-condition is led into the cooling tower and pumped to a sprinkler. The sprinkler is spreading the water into a heat exchange surface, which is cooled by the air flow and therefore also cooling the water. The cooled water is dropping into the water reservoir and will be pumped back in to the water cycle. The air flow through the ACCT releases a massive amount of aerosolized water. (Inspired by hvactutorial.wordpress.com)
