# Supplementary material for: Air-conditioner cooling towers as complex reservoirs and continuous source of Legionella pneumophila infection evidenced by a genomic analysis study in 2017, Switzerland
Source: Euro Surveill. 2019 Jan 24;24(4):1800192. doi: 10.2807/1560-7917.ES.2019.24.4.1800192 (PMC6351994; doi:10.2807/1560-7917.ES.2019.24.4.1800192)
Supplement: Supplementary Figure S4 [file 1800192_SupplementaryFigureS4.pdf]

This supplementary material is hosted by Eurosurveillance as supporting information alongside the article “Air-conditioner cooling towers as complex reservoirs and continuous source of Legionella pneumophila infection evidenced by a genomic analysis study in 2017, Switzerland” on behalf of the authors who remain responsible for the accuracy and appropriateness of the content. The same standards for ethics, copyright, attributions and permissions as for the article apply. Eurosurveillance is not responsible for the maintenance of any links or email addresses provided therein.

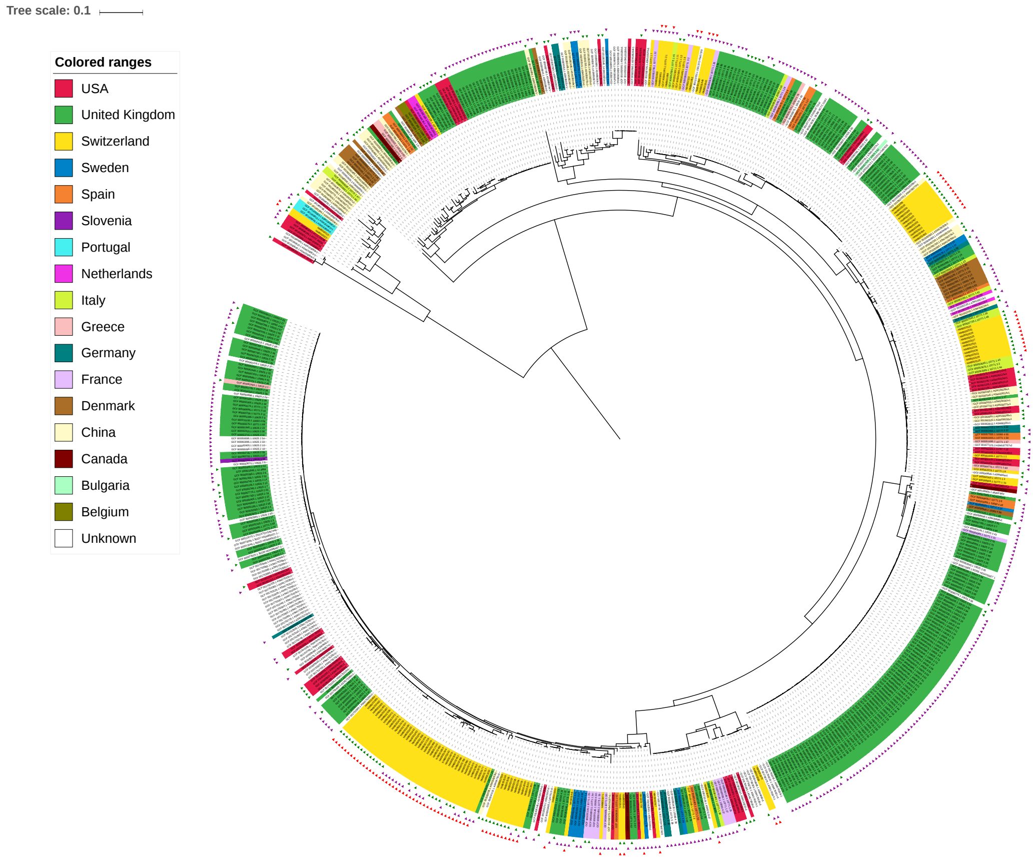

Figure S4: Global context of Lp strains. The branch lengths represent the phylogenetic distance between the genomes. The colors of the labels indicate the countries in which the isolates were extracted (17 countries). The environmental isolates are marked with a green triangle and the clinical isolates with purple triangle. The Isolates of this study are labeled with a red triangle.
